# A Low Glycemic Index Mediterranean Diet Combined with Aerobic Physical Activity Rearranges the Gut Microbiota Signature in NAFLD Patients

**DOI:** 10.3390/nu14091773

**Published:** 2022-04-23

**Authors:** Francesco Maria Calabrese, Vittoria Disciglio, Isabella Franco, Paolo Sorino, Caterina Bonfiglio, Antonella Bianco, Angelo Campanella, Tamara Lippolis, Pasqua Letizia Pesole, Maurizio Polignano, Mirco Vacca, Giusy Rita Caponio, Gianluigi Giannelli, Maria De Angelis, Alberto Ruben Osella

**Affiliations:** 1 Department of Soil, Plant and Food Science, University of Bari Aldo Moro, 70126 Bari, Italy; mirco.vacca@uniba.it (M.V.); maria.deangelis@uniba.it (M.D.A.); 2 National Institute of Gastroenterology “S. de Bellis” Research Hospital, Castellana Grotte, 70013 Bari, Italy; vittoria.disciglio@irccsdebellis.it (V.D.); isabella.franco@irccsdebellis.it (I.F.); paolo.sorino@irccsdebellis.it (P.S.); catia.bonfiglio@irccsdebellis.it (C.B.); antonella.bianco@irccsdebellis.it (A.B.); angelo.campanella@irccsdebellis.it (A.C.); tamara.lippolis@irccsdebellis.it (T.L.); letizia.pesole@irccsdebellis.it (P.L.P.); maurizio.polignano@irccsdebellis.it (M.P.); giusy.caponio@irccsdebellis.it (G.R.C.); gianluigi.giannelli@irccsdebellis.it (G.G.)

**Keywords:** NAFLD, gut microbiota, lifestyle intervention, Mediterranean diet, physical activity

## Abstract

Non-alcoholic fatty liver disease (NAFLD) is the most common liver disease, and its prevalence worldwide is increasing. Several studies support the pathophysiological role of the gut–liver axis, where specific signal pathways are finely tuned by intestinal microbiota both in the onset and progression of NAFLD. In the present study, we investigate the impact of different lifestyle interventions on the gut microbiota composition in 109 NAFLD patients randomly allocated to six lifestyle intervention groups: Low Glycemic Index Mediterranean Diet (LGIMD), aerobic activity program (ATFIS_1), combined activity program (ATFIS_2), LGIMD plus ATFIS_1 or ATFIS2 and Control Diet based on CREA-AN (INRAN). The relative abundances of microbial taxa at all taxonomic levels were explored in all the intervention groups and used to cluster samples based on a statistical approach, relying both on the discriminant analysis of principal components (DAPCs) and on a linear regression model. Our analyses reveal important differences when physical activity and the Mediterranean diet are merged as treatment and allow us to identify the most statistically significant taxa linked with liver protection. These findings agree with the decreased ‘controlled attenuation parameter’ (CAP) detected in the LGIMD-ATFIS_1 group, measured using FibroScan^®^. In conclusion, our study demonstrates the synergistic effect of lifestyle interventions (diet and/or physical activity programs) on the gut microbiota composition in NAFLD patients.

## 1. Introduction

Non-alcoholic fatty liver disease (NAFLD) is the most common metabolic-associated fatty liver disease. Moreover, the incidence and prevalence of NAFLD that affects up to nearly one third of adults in the general population of Western countries are rapidly rising worldwide [1,2].

NAFLD is characterized by the presence of at least 5% of hepatic steatosis without evidence of the secondary causes of hepatic fat accumulation, including excessive alcohol consumption, chronic hepatitis C virus (HCV) infection, autoimmune hepatitis, or congenital hepatic disorders [3,4]. NAFLD encompasses a wide spectrum of liver diseases, ranging from a simple accumulation of liver fat to a more complex clinical picture characterized by the development of non-alcoholic steatohepatitis (NASH), which can potentially progress to fibrosis, cirrhosis, and eventually hepatocellular carcinoma (HCC) [5].

Importantly, NAFLD-related liver failure is one of the major causes of liver transplantation in the Western world [6]. For this reason, research efforts are crucial to implement early diagnosis and interventions to prevent or reverse the deleterious consequences of advanced NAFLD.

A rapidly growing body of evidence supports the pathophysiological role of the gut–liver axis signaling pathways, modulated by gut microbiota-related mechanisms, in the onset and progression of NAFLD [7].

In the absence of approved pharmacological therapies for NAFLD treatment, the European clinical guidelines recommend lifestyle interventions, including dietary changes and regular exercise, as the best therapeutic strategy for the management of NAFLD patients [8,9]. Accordingly, diet may improve NAFLD by reducing liver fat and insulin resistance [10,11]. Moreover, physical activity has been shown to achieve weight loss and improve liver functionality [12,13]. Recent studies suggest that the modification of the gut microbiota may be a novel strategy to prevent or treat NAFLD [14,15]. Accordingly, various dietary regimens and regular physical activity have been shown to induce changes in the composition of gut microbiota, in association with a rapid improvement to insulin sensitivity and lipid metabolism [16,17].

The gut microbiota alterations associated with NAFLD and NASH, which cause a state of dysbiosis, involve several microbial taxa. NAFLD patients harbor a higher prevalence of bacterial translocation to the small intestine, increasing both intestinal permeability and circulating endotoxin levels [18]. The increased concentration of circulating endotoxins triggers an inflammatory and fibrotic hepatic response [19].

Bile acid, synthesized from cholesterol in the liver, has been demonstrated to play a crucial role in the pathophysiology of NAFLD. The primary bile acids could be deconjugated and dehydroxylated by the gut microbiota into secondary bile acids, which are reabsorbed and returned to the liver through the portal vein [20,21]. Some bacterial species in the colon can intervene in this metabolism, causing changes in the abundance and composition of circulating bile acids. Higher serum levels of bile acids have been found in NAFLD and NASH patients, compared to healthy individuals, including secondary bile acids [22]. Short-chain fatty acids (SCFAs), which are anaerobic fermentation products generated by the intestinal microbiota from non-digestible carbohydrates, are transferred to the liver via the portal circulation and act as precursors for hepatic lipogenesis or gluconeogenesis. SCFAs regulate immune homeostasis and influence hepatic metabolism, and regulate host metabolism through an epigenetic mechanism [23]. The long-term intake of an unhealthy diet (i.e., enriched in saturated fats or fructose) can predispose patients to gut microbiota dysbiosis, and the subsequent disruption of the intestinal barrier function and of immune homeostasis. In addition, SCFAs can decrease inflammatory responses, oxidative damage, and lipogenesis in the liver tissue. Therefore, intestinal microbes are considered to be the key element regulating the pathological process of NAFLD. Noticeably, starchy food products, such as grains and legumes, especially abundant in the Mediterranean diet, are carriers of less fermentable insoluble fibers that elicit protective properties, including the glycemic control, postprandial glycemia [24] and liver fat [25], mechanistically linked to BCAA uptake [26] and bile acid metabolism [27], insulin sensitivity [28] and various long-term outcomes [29]. 

The effects of two different physical activity programs (aerobic activity and resistance training), two different diets (Control Diet based on CREA-AN (INRAN) guidelines and Low Glycemic Index Mediterranean Diet (LGIMD)) and their combination, were previously investigated in a cohort of NAFLD patients [30]. This previously analyzed subject cohort showed a significant NAFLD score reduction after 45 days of treatment.

Based on this evidence and in line with the existing literature background, our first aim is to investigate how different lifestyle interventions, including diet and physical activity, can impact the gut microbiota composition in NAFLD patients after 45 and 90 days of treatment. The statistical approach we adopt to analyze our NAFLD patient cohort reveals statistically significant differences in terms of microbiota taxa abundances, mostly evident in the group allocated to the combined intervention consisting of aerobic physical activity and a Mediterranean dietary regimen. Secondly, starting from the 16S data obtained, we predict the abundance of microbiota metabolic pathways and identify the only one that is statistically significant.

## 2. Materials and Methods

### 2.1. Subjects and Lifestyle Interventions

Details of the study design have been published elsewhere [30,31]. Briefly, the NUTRIATT study was a randomized clinical trial (RCT) (https:www.clinicaltrials.gov (accessed on 15 October 2021, NCT02347696) based on lifestyle interventions, conducted in South Italy from March 2015 to January 2020. In this study, subjects with NAFLD in both hospital and general practitioners’ settings were enrolled. The inclusion criteria for this study were: (1) body mass index (BMI) ≥ 25 kg /m^2^; (2) age >30 years old <60; and (3) moderate or severe NAFLD. The exclusion criteria included: (1) overt cardiovascular disease and revascularization procedures; (2) stroke; (3) clinical peripheral artery disease; (4) current treatment with insulin or oral hypoglycemic drugs; (5) fasting glucose >126 mg/dL, or casual glucose > 200 mg/dL; (6) more than 20 g/day of alcohol intake; (7) medical conditions that could affect participation in a nutritional intervention study; and (8) people following a special diet, involved in a weight loss program, who had experienced recent weight loss, or were unable to follow a diet for religious or other reasons. Details about lifestyle interventions, enrolment and sample size estimation have been previously described [24]. In total, 166 subjects were randomized to 6 study categories, as follows: (1) Control Diet (INRAN) based on CREA-AN [32]; (2) Low Glycemic Index Mediterranean Diet (LGIMD) [33]; (3) physical activity aerobic program (ATFIS_1); (4) physical activity combined program (aerobic activity and resistance training) (ATFIS_2); (5) LGIMD plus ATFIS_1; and (6) LGIMD plus ATFIS_2. Stool samples were collected after 45 and after 90 days of treatment. 

### 2.2. The CAP Score Measurement

The controlled attenuation parameter (CAP) score was used to detect and quantify hepatic steatosis. Specifically, the CAP parameter measures the degree of ultrasound attenuation due to hepatic fat at the standardized frequency of 3.5 MHz through a vibration-controlled elastography (VCTE) and was quantified as dB/m. This procedure was implemented on FibroScan^®^ (Echosens, Paris, France) and was recorded for each subject. Following the recommended cut-off values, NAFLD was classified as absent (<248), mild (248–267), moderate (268–279) and severe (≥280) [34]. All the subjects provided written informed consent to participate. The study was conducted according to the Declaration of Helsinki and approved by the Ethics Committee (Prot. n. 10/CE/De Bellis, 3 February 2015).

### 2.3. 16S rRNA Gene Amplicon Sequencing

Fresh fecal samples were collected from all subjects. Total bacterial metagenomic DNA was extracted from stool samples using the QIAamp FAST DNA Stool Mini Kit (Qiagen, Hilden, Germany), according to the manufacturer’s instructions. The final yield and quality of extracted DNA were determined using a NanoDrop ND-1000 spectrophotometer (Thermo Scientific, Waltham, MA, USA) and Qubit Fluorometer 1.0 (Invitrogen Co., Carlsbad, CA, USA). The 16S metagenomic analyses were performed at Genomix4life S.R.L. (Baronissi, Salerno, Italy). Specifically, 16S amplification was performed with the primers, Forward: 5′-CCTACGGGNGGCWGCAG-3′ and Reverse: 5′-GACTACHVGGGTATCTAATCC-3′ [35], which target the hypervariable V3 and V4 regions of the 16S rRNA gene. Each PCR reaction was assembled according to the Metagenomic Sequencing Library Preparation (Illumina, San Diego, CA, USA). A negative control was included in the workflow, consisting of all the reagents used during the sample processing (16S amplification and library preparation), but not containing the sample, to avoid contamination. The resulting libraries were quantified using a Qubit fluorometer (Invitrogen Co., Carlsbad, CA, USA) and pooled to an equimolar amount of each index-tagged sample at a final concentration of 4 nM, including the Phix Control Library. The pooled samples were subjected to cluster generation and sequenced on the MiSeq platform (Illumina, San Diego, CA, USA) in a 2 × 300 paired-end format.

### 2.4. Bioinformatic Analyses

16S sequencing-derived fastQ files were checked for quality using FastQC software [36]. In silico bioinformatics analyses, including denoizing, taxa assignment and alpha and beta diversity, relied on the QIIME2 [37] microbiome platform (version 2020.8). More specifically, the QIIME plugin q2-deblur (https://github.com/qiime2/q2-deblur (accessed on 15 October 2021) was used for the 16S denoizing step and Shannon entropy and Faith’s PD were computed on the significance obtained using ad hoc available plugins. The SILVA 138 SSU database (https://www.arb-silva.de/documentation/release-138/ (accessed on 15 October 2021) was used to infer the taxonomy starting from the ASV table. All the computed intermediate and final outputs not included, either as main documents or as Supplementary Materials, are available upon request, and will be provided in the qzv QIIME2 format.

### 2.5. Statistical Analyses

In order to assess the beta diversity occurring within our population, a first inspection of the taxa abundance distribution was conducted by means of Principal Component Analysis (PCA) in the R environment using the ‘FactoMineR’ package (Multivariate Exploratory Data Analysis and Data Mining) version 2.4 available in the CRAN repository (https://cran.r-project.org/web/packages/FactoMineR/index.html (accessed on 15 October 2021)). The evidence of clustering among our samples was inspected by means of the discriminant analysis of principal components (DAPC), a multivariate analysis based on the selection of few synthetic variables (linear combinations of the original ones). Specifically, in a first step, the DAPC was run on the taxa abundance matrix without superimposing any metadata grouping condition and using the ‘find.clusters’ clustering algorithm. Subsequently, according to the obtained metadata information, the assigned dietary/physical activity intervention group of each sample was set as the a priori condition. The prior and posterior membership probabilities were computed and graphically translated using the ‘assignplot’ function within the adegenet R package v2.1.1 (https://cran.r-project.org/web/packages/adegenet/index.html (accessed on 15 October 2021). This allowed us to calculate the proportions of successful reassignments. To assess the weight of each variable, the taxa contributing most strongly to cluster separation were computed and plotted using the ‘assignplot’ function within the R adegenet package.

#### 2.5.1. Multivariable Fitting by the MaAslin2 R Package

The multivariable associations among 16S rRNA gene data abundances at different taxonomic levels, based on the microbial NAFLD sample meta-omics features, were computed using the general linear model implemented in the ‘MaAsLin 2′ R package (Microbiome Multivariable Association with Linear Models). After multiple test corrections, the significant tossed-out taxa resulting from pair-group comparisons were plotted singly as bar plots [38].

#### 2.5.2. Inferring Metacyc Metabolic Pathways from 16S ASV Data

Per-sample metabolic pathway predictions from 16S rRNA marker gene data were obtained using Picrust2 software, which was run as a plugin within the QIIME2 library. Per-sample MetaCyc pathway abundances were used as inputs for a two-sided Welch test between the groups. A multiple test correction was conducted with Benjamini–Hochberg (q < 0.05) using STAMP software [39].

## 3. Results

### 3.1. The Characteristics of the Participants

A total of 166 subjects were assessed for eligibility for the present trial; 17 subjects were excluded because they did not satisfy all the inclusion criteria and 5 for various other reasons. The loss of 5 subjects occurred before the sampling follow up. A total of 30 subjects were excluded because they failed to return their feces collections. Finally, 109 subjects were evaluated in this study, randomly allocated to one of the six groups: (1) INRAN group (*n* = 17); (2) LGIMD group (*n* = 19); (3) ATFIS_1 group (*n* = 19); (4) ATFIS_2 group (*n* = 18); (5) LGIMD plus ATFIS_1 group (*n* = 17); and (6) LGIMD plus ATFIS_2 group (*n* = 19). The trial flowchart is shown in Figure S1. The characteristics of the included participants are summarized in Table 1.

As expected, the age–sex distribution of the population under study reflected the age–sex distribution of the NAFLD condition in the population [34]; 53% were men. The mean age was 52.81 years (±9.29), while 12.5% of the subjects were younger than 40 years old. All subjects were overweight (BMI ranging between 25 and 29.9) or obese (BMI over 30). All the parameters considered were equally distributed among the groups at baseline, with the only exception of NAFLD severity. In fact, more subjects had severe than moderate NAFLD, and the CAP value was over 323 dB/m in all groups. In fact, NAFLD severity was not used to stratify our patient cohort.

### 3.2. Sequencing Statistics and Statistical Approach

**Figure 1:**
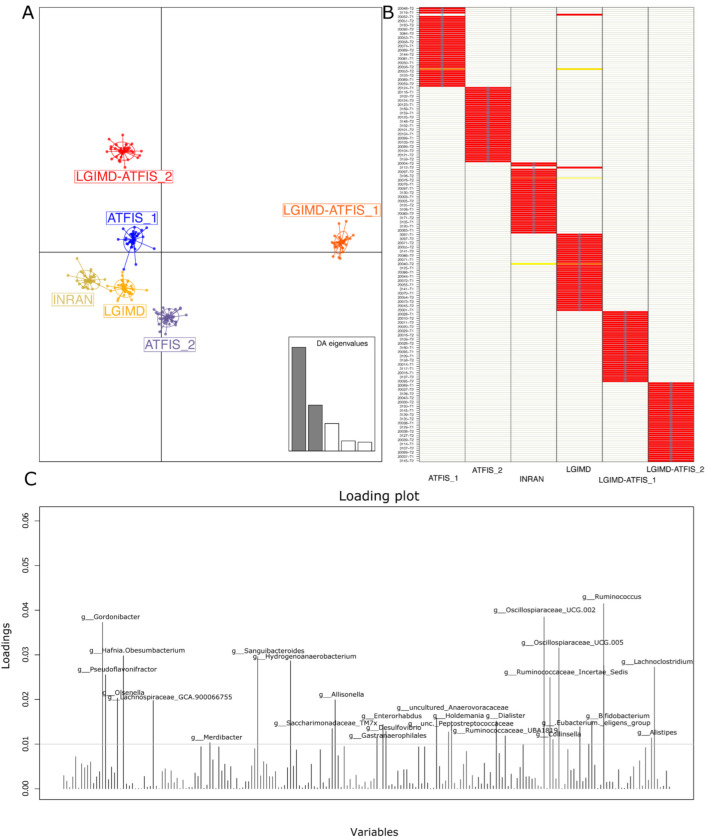
DAPC analysis based on the adegenet R package: (**A**) DAPC plot obtained by superimposing samples on the prior group assignment with the screeplot of used discriminant analysis (DA) eigenvalues (two out of five in grey colour) reported in the bottom right of the panel; (**B**) proportions of successful reassignments: heat colors represent membership probabilities (red = 1, white = 0, orange/yellow = non completely succeeded reassignment) and blue crosses represent the DAPC prior cluster; and (**C**) loading DAPC plot reporting the genera that best highlighted the cluster separation. The variables that contributed the most to the DAPC loading plot are over the 0.01 threshold.

The demux filtered statistics on the whole set composed of 215 samples yielded a number of total retained reads ranging from approximately 43 K up to 321 K, whereas the number of reads that were truncated, or that exceeded the maximum ambiguous bases in the Deblur filtering, was negligible (Supplementary Materials: Tables S2–S4). Taxa assignments with relative abundances were reported for all the 6 taxonomic levels (Supplementary Materials: Tables S5–S10). The Shannon and Faith’s PD metrics were computed in QIIME2 and subjected to the Kruskal–Wallis pairwise test to find the statistically significant differences among the groups. Both metrics were concordant in depicting a non-significant variation of the alpha diversity among the compared six groups (Figure S2 and Table S11). When assessing the variables that contributed the most to stratify the samples, principal component analysis based on taxa relative abundances revealed a homogeneous distribution of the samples, without any stratification. No separation in terms of the linear distance was observed either at the family (data not shown) or the genus level (Figure S3). As a second approach, discriminant analyses of the principal components (DAPC) were run to infer possible sample stratifications. The samples at the two follow-up times (T1 and T2) were used without superimposing the group assignment. Specifically, based on the genus abundances, in order to identify the best fitting number of clusters for the NAFLD samples, the ‘find.cluster’ function within the adegenet R package was used. The nested k-mean algorithm finds a given number of groups maximizing the variation between the groups. The minimum elbow point reached in the Bayesian Information Criterion (BIC) curve indicated the best clustering solution corresponding to the prior six sample groups (Figure S4 panel A). The scatter DAPC result was obtained by plotting the ‘find.cluster’ function output object (Figure S4 panel B). Six possible clusters were identified and the location of one of them, number six, was far from the others (Figure S4 panel B). A second DAPC plot was then obtained by superimposing the samples on their group, among the six a priori assigned groups (Figure 1). The plot demonstrated that the combination of the aerobic physical activity program with the Mediterranean diet (LGIMD-ATFIS_1) resulted in a cluster located far from the other clusters. The other clusters, including diet and physical activity non-combined interventions, were located across the second and third DAPC quadrants, whereas the LGIMD-ATFIS_2 group deviated from this partially aggregated cluster. Except for only two discrepancies (one in the T1 and one in the T2 sample groups), the membership probability was verified for all the T1 and T2 samples.

**Figure 2:**
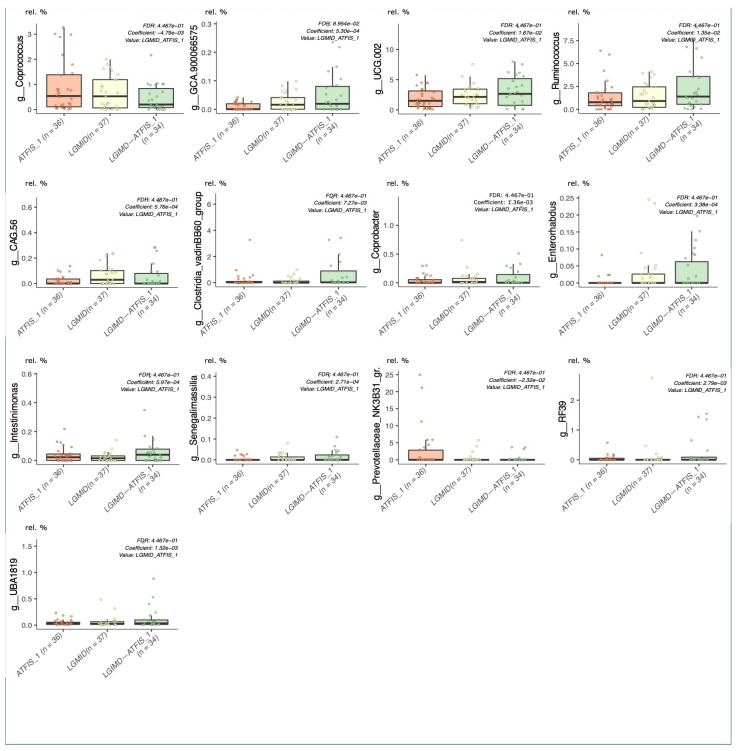
Maaslin2 associations in the single versus combined intervention groups. Aerobic physical activity (ATFIS_1), Mediterranean diet (LGIMD), and the combined LGIMD-ATFIS_1 intervention groups were compared by means of the linear regression model (Maaslin2), determining the multivariable associations between the phenotypes. Taxa relative abundances were reported on the Y axis.

Considering the cumulative effect of the variables on the cluster separation, well described by the DAPC analysis, those taxa that were over the threshold in the loading plot graph were inspected for their relative abundances in each of the considered groups, and graphically rendered as single boxplot interquartile distributions. Although these taxa were the major contributors to the above-described DAPC analysis, not all of them showed a clear trend in describing the separation of the LGIMD-ATFIS_1 cluster. When comparing all the groups, 19 genera did not show a clear tendency (data not shown), whereas the other 9 (Figure S5) had a different, greater distribution of LGIMD-ATFIS_1. Six genera had increased relative abundances in the LGIMD-ATFIS_1 samples, when compared to the other groups, i.e., *Ruminococcus*, *Oscillospiraceae-UCG002*, *Oscillospiraceae-UCG005*, *Dialister*, *Alistipes*, and *Eubacterium eligens* groups, whereas *Collinsella* showed a decreasing and opposite trend. Because of the lack of clear differences between the LGIMD-ATFIS_1 vs. both the non-combined LGIMD and the ATFIS_1 groups, the statistically significant differences in terms of taxa abundances were evaluated in more depth using the Maaslin2 general linear model. When the aerobic physical activity program, the Mediterranean diet, and the combination of these regimens were tested for association, nine genera were found to be statistically significant. Specifically, *Ruminococcus*, *Lachnospiraceae_GCA900066575*, *Clostridia VadinBB60 group*, *Enterorabdus*, *Coprobacter*, *UCG002* (*Oscillospiraceae*), *Intestinimonas*, and *Ruminococcaceae_g_UBA1819* were higher in LGIMD-ATFIS_1, whereas *Coprococcus* showed an opposite trend (Figure 2).

**Figure 3:**
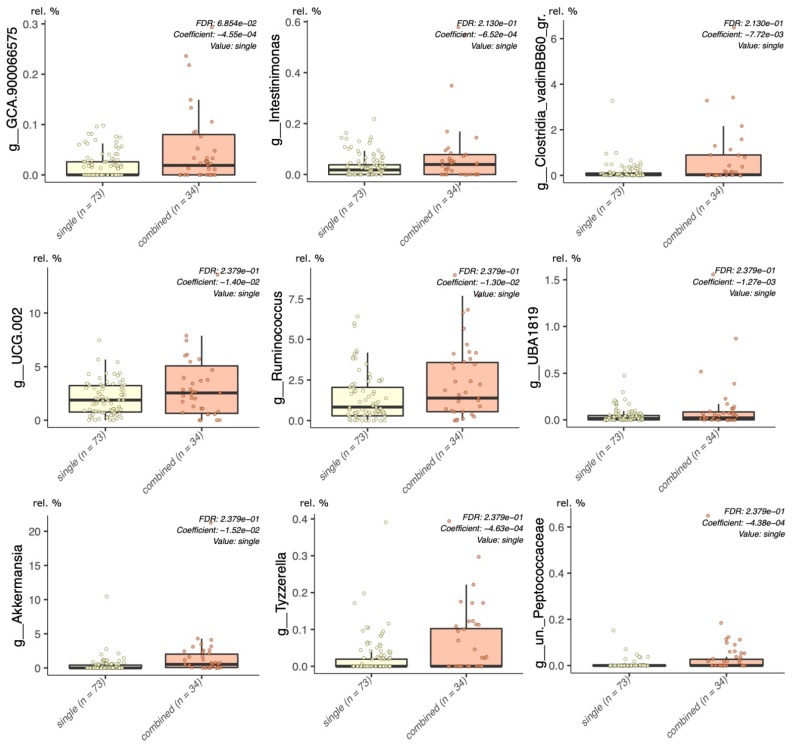
Maaslin2 model, single versus combined groups. Grouped single (ATFIS_1 and LGIMD) interventions were compared with the combined (LGIMD-ATFIS_1) group, setting the allocation to each of the three groups as the random effect in the linear model.

When the single lifestyle interventions (LGIMD and ATFIS_1) were merged into a single group and statistically significant associations were sought by setting the intervention group as a random effect, the genera *Ruminococcus*, *Oscillospiraceae_g_UCG002*, *Intestinimonas, Ruminococcaceae_g_UBA1819, Lachnospiraceae_GCA900066575*, and *Clostridia VadinBB60 group* were confirmed to have a statistically significant lower abundance, when compared to the LGIMD-ATFIS_1 combined group (Figure 3). Moreover, three other genera, i.e., *Akkermansia*, *Tyzzerella*, and *uncultured_Peptococcaceae,* were found to be higher in LGIMD-ATFIS_1.

The Metacyc-predicted metabolic pathways (inferred from the QIIME ASV table abundances) were inspected using a two-group Welch test, and only those that were statistically significant (*q* < 0.05) after multiple test corrections (BH) were considered. As reported in Figure S6, only one pathway was statistically significant, when comparing the ATFIS_1 and LGIMD-ATFIS_1 groups. Specifically, in this last comparison, the CMP–legionaminate biosynthesis was found to be less abundant in those patients following the regimen that included anaerobic physical activity and the Mediterranean diet.

## 4. Discussion

The gut microbiota and its metabolites were demonstrated to play a role in the pathophysiology of NAFLD through the gut–liver axis [40]. Several studies have shown the potential role of dietary interventions in modulating the intestinal microbiota composition [41]. Moreover, physical activity has been demonstrated to impact the composition and functionality of the gut microbial population, yielding potential health benefits [42] on the gut–liver axis. Given the correlation between gut microbiota and NAFLD, in the present study, we sought to evaluate the effects of different lifestyle interventions, including two different diets (LGIMD and INRAN), alone or in combination with physical activity programs (aerobic activity and resistance training), on the gut microbiota plasticity. The absence of statistically significant differences in the alpha diversity detected values among the groups emphasized that balanced diets (LGIMD and INRAN), which can guarantee an optimal fiber and macro- and micronutrient intakes, are sufficient to determine a highly resilient gut microbiota [43]. 

Starchy food products abundant in the Mediterranean diet are carriers of less fermentable insoluble fibers that presented protective and important properties for immune homeostasis and the inflammatory response. Specific bacterial strains are capable of metabolizing unabsorbed carbohydrates; this activity, as in the case of obese patients (as the great majority of NAFLD subjects), is a consequence of the uptake of bioavailable SCFAs that leads to an additional availability of energy sources [44]. Fermentable dietary fibers also proved to exert anti-oxidation, anti-inflammatory and anti-tumor activities [45]. High-fat diets can modulate gut microbiota whose dysbiosis was highly associated with NAFLD [46].

In a previous work, we explored the differences of samples at the baseline from those after 45 and 90 days of treatment [30]. Here, the group-specific differences at the genus level were obtained by focusing on the dietary and physical activity groups after treatment.

Some interesting differences emerged from the DAPC analysis. The multivariate statistical approach that we adopted works by maximizing the variance between groups, in order to trace the major contributing differences among the groups. The absence of a physical activity program clearly determined few differences between the LGIMD and INRAN diets, suggesting that both interventions could be considered as useful nutritional therapies in managing NAFLD-induced dysbiosis. The same discriminant analysis succeeded in differentiating the ATFIS-1 from the ATFIS-2 intervention group, and this evidence was strengthened when the physical activity program and the Mediterranean diet were combined. When observing the DAPC membership probability, the match between the a priori versus the posterior assignments confirmed that almost all samples did actually belong to the relative group (only 2 out of 109 subjects were exceptions). The inspection for the variables most strongly influencing the group clustering obtained revealed a batch of 28 microbial genera that were then evaluated further. Many of the identified taxa had been recently assessed in another work that focused on the different regimens of physical activity in NAFLD patients [47]. However, in our patient cohort, we disclosed how some of these taxa were poorly representative of the entire gut microbiota community. Furthermore, when the differences in taxa relative abundance were inspected among the group pairs, the MaAsLin2 regression model showed a non-exclusive contribution of the LGIMD-ATFIS_1 group only.

The combination of aerobic physical activity and the Mediterranean diet, measured through the CAP score in our cohort of NAFLD patients, had already been shown to reduce the severity of liver steatosis. This is indicative of an improved pathologic status in the LGIMD-ATFIS_1 group of patients. It is noteworthy that the LGIMD or ATFIS_1 regimens, considered alone, were sufficient to determine an improved trend [30]. Based on this evidence, we applied the MaAsLin2 regression model with a specific focus on the single comparison of LGIMD-ATFIS_1 against both LGIMD and ATFIS_1. The statistical model identified nine statistically significant taxa, which were all enriched in LGIMD-ATFIS_1 (labeled as ‘combined’). With the only exception of *Akkermansia*, which hierarchically belongs to the Verrucomicrobia phylum, all the other significant genera are Firmicutes sub-taxa. The relevance of this result is in line with published studies reporting a decrease in Firmicutes, positively associated with NAFLD progression [21,48]. This evidence is also confirmed by a recent meta-analysis [49] and other research studies [50,51], all underlining a clear lack of *Ruminococcaceae* in NAFLD patients. Importantly, this family relative abundance increased in our LGIMD-ATFIS_1 category.

In NAFLD, *Ruminococcaceae* contribute to liver protection by improving the gastrointestinal barrier integrity, modulating the gut microbiome [52,53,54]. Although the *Ruminococcaceae* and *Lachnospiraceae* families both contribute to the fiber digestion metabolism, the role of taxa members belonging to the latter family seems to be controversial in NAFLD patients [49,55]. Moreover, increasing *Lachnospiraceae* have been constantly demonstrated during progression from NAFLD to NASH [56]. In addition to the liver injury characterizing NAFLD patients, another critical issue is bile acid (BA) metabolism. Several works discussed the contribution of the gut microbiota in BA singling and recycling [57]. According to this scenario, it is widely recognized that perturbations in microbiota composition, commonly referred to using the term dysbiosis, might also negatively affect the gut microbiota–host BA axis [58]. From this perspective, various research groups specifically searched for differences occurring in BA metabolism and compared dysbiotic vs. healthy microbiota [59]. Mullish et al. found a cluster of bacterial families positively linked with secondary bile acids; this cluster encompassed *Bacteroidaceae*, *Lachnospiraceae*, *Ruminococcaeceae*, and *Oscillospiraceae* [59]. Moreover, indole-3-propionic acid (IPA) has been associated with liver inflammation and fibrosis and its circulating levels reveal how this molecule diminished in patients with liver fibrosis, where the Mediterranean diet increases its microbial synthesis [60]. In confirming that IPA can regulate liver fibrosis in humans, other researchers argued about the higher mRNA levels of genes that are part of the metabolic pathways fundamental to hepatic stellate cell activation and fibrosis signaling [61] (doi: 10.3390/nu13103509).

Most of the taxa we found that were increased in LGIMD-ATFIS_1 belong to the above-mentioned families. Specifically, among the nine LGIMD-ATFIS_1 statistically significant genera, 2 were sub-taxa of *Lachnospiraceae* (*GCA900066575* and *Tyzzerella*), 2 of *Ruminococcaeceae* (*Ruminococcus* and *UBA1819*), and 2 of *Oscillospiraceae* (*UCG002* and *Intestinimonas*).

Lastly, as previously mentioned, the only genus not included in the Firmicutes phylum to exhibit an increased abundance in LGIMD-ATFIS_1 was *Akkermansia*. This taxon was proposed as a candidate probiotic in the treatment of various diseases [62]. *Akkermansia* and *Clostridum* were both found to have a higher abundance in the LGIMD-ATFIS_1 group and, as evidenced in the literature, species belonging to both these genera have proven to take part in the synthesis of IPA [63].

Although the consumption of symbiotic bacteria, such as *Akkermansia muciniphila*, was used to ameliorate the NAFLD patients’ conditions [64], the role of *Akkermansia* (often found increased in NAFLD patients) is still under discussion [49]. The contribution of *Akkermansia* in favoring gut well-being is largely supported in the literature, while many organisms living in the gut could exert adverse activities. Therefore, considering that, in our area of study, LGIMD-ATFIS_1 patients followed both an optimal diet and physical activity regimen, and that *Akkermansia* was detected in lower abundance values in single (combining LGIMD plus ATFIS_1 patients) versus the combined group (LGIMD-ATFIS_1 patients), we may speculate on its contribution as an additional beneficial outcome of the adoption of an LGIMD-ATFIS_1 regimen. To understand whether the contribution of taxa may result in effective metabolic differences among the investigated groups, the complete matrix of genus abundances was used to predict the metabolic pathways. The only statistically significant result was relative to the CMP–legionaminate biosynthesis I pathway, which discriminated the LGIMD-ATFIS_1 versus the ATFIS_1 group. 

Various enzymes are involved in the CMP–legionaminic acid pathway producing a bacterial analog of sialic acid [65]. In humans, sialic acid-binding immunoglobulin-like lectins are immune-modulating proteins that are differentially expressed on hematopoietic cells; peripheral natural killer (NK) cells are less frequent in NAFLD patients [66]. Serum levels of lectin sSiglec-7 were significantly higher in NAFLD versus healthy patients [66]. The CMP–legionaminate biosynthesis pathway was significantly higher in the ATFIS_1 group, compared to the combined LGIMD-ATFIS_1 group. The lower predicted values in this pathway led us to speculate about a possible protective effect of this analog obtained by the combination of diet and physical activity. According to this hypothesis, the microbial analog would interact by causing a slowdown of the immune-regulating proteins acting synergically with NK cells, critical components of the innate immune system.

The statistical approach applied here allowed us to explore specific microbiota rearrangements, due to different tested dietary and physical activity regimens. The improved health status benefits from specific genus abundance shifts that fit the decreased CAP parameter.

Although the synergistic effect resulting from the combination of diet and physical activity on our large subject cohort proved to improve the health status of NAFLD patients, the lack of metabolite profiles and the deep characterization of the metabolic pathways in which these metabolites are involved is the strongest limitation of the present study. An ad hoc designed metabolomics experiment would allow for a better understanding of how these mixed interventions impact NAFLD patient metabolism.

Among the study strengths it is worth including the study design, the structured nature of both diet and physical activity programs, the measured compliance to both interventions as well as their controlled application. Moreover, the sample size was estimated to account for the effect size, power and the correlation among measures on the same subject. Furthermore, a well-validated assessment of the outcome, such as FibroScan^®^, was implemented. The applied intention-to-treat analytical strategy prevented the design from introducing bias relating to non-adherence to the protocol of the prognosis; therefore, this RCT provided an unbiased assessment of treatment efficacy [67]. Moreover, it has to be considered that, in our sampling area, the most prevalent dietary pattern was the local version of the Mediterranean diet; thus, a dilution bias could be present. Another limitation may be the duration of the intervention, which prevented a wide application in the clinical field. However, the objective of the study was to estimate the effect of the intervention that was designed in order to establish its efficacy.

## 5. Conclusions

Our results highlight the contribution resulting from the synergistic effect of lifestyle interventions (diet and/or physical activity programs) on the composition of the gut microbiota in NAFLD patients. In the first instance, we found that nutritional therapies based on dietary interventions contributed to reduce the dysbiosis characterizing NAFLD patients, increasing the resilience of microbial communities inhabiting the gut. Furthermore, the adoption of an aerobic exercise program, in combination with the Mediterranean diet (LGIMD-ATFIS_1), was able to further ameliorate specific ratios of microbes, as also evidenced by the CAP parameter used to measure steatosis levels. Further studies are needed to investigate, in greater depth, the genomic potential and expression level of microbes that contribute the most to the metagenomics and metatranscriptomics levels. 

## Figures and Tables

**Table 1:** Anthropometric and clinical features of the NAFLD patients.

	Working Arms					
Variables	INRAN	LGIMD	ATFIS_1	ATFIS_2	LGIMD-ATFIS_1	LGIMD-ATFIS_2
# NAFLD patients	17	19	19	18	17	19
Age (years) *	56.03 (8.37)	56.76 (9.18)	52.32 (7.46)	53.38 (6.27)	50.54 (10.86)	48.44 (11.54)
Age categories (years) **						
<45	2 (9%)	2 (9%)	3 (13%)	3 (13%)	6 (26%)	7 (30%)
45–54	5 (14%)	3 (8%)	10 (28%)	9 (25%)	4 (11%)	5 (14%)
55–59	4 (17%)	7 (30%)	4 (17%)	2 (9%)	3 (13%)	3 (13%)
≥60	6 (23%)	6 (23%)	2 (8%)	4 (15%)	4 (15%)	4 (15%)
Gender **						
Female	4 (8%)	9 (18%)	8 (16%)	10 (20%)	6 (12%)	13 (26%)
Male	13 (23%)	8 (14%)	11 (19%)	8 (14%)	11 (19%)	6 (11%)
BMI *	33.45 (4.37)	33.50 (6.44)	30.54 (4.11)	31.81 (3.46)	32.99 (5.19)	32.28 (4.38)
BMI categories **						
25–29	3 (9%)	8 (24%)	7 (21%)	6 (18%)	5 (15%)	4 (12%)
30–35	10 (20%)	4 (8%)	10 (20%)	10 (20%)	8 (16%)	9 (18%)
>35	4 (19%)	6 (29%)	2 (10%)	2 (10%)	4 (19%)	3 (14%)
CAP (dB/m) *	348.35 (38.34)	341.34 (20.24)	325.46 (19.83)	332.71 (28.66)	323.76 (33.20)	328.22 (40.32)
Grading of liver Steatosis **						
Absent	0 (0%)	1 (13%)	2 (25%)	3 (38%)	1 (13%)	1 (13%)
Mild	1 (6%)	5 (28%)	3 (17%)	3 (17%)	3 (17%)	3 (17%)
Moderate	10 (21%)	5 (11%)	9 (19%)	9 (19%)	7 (15%)	7 (15%)
Severe	6 (18%)	6 (18%)	5 (15%)	3 (9%)	6 (18%)	8 (24%)
Tryglicerides (mmol/L) *	1.18 (0.62)	1.79 (1.13)	1.81 (0.78)	1.63 (0.97)	1.94 (1.38)	1.50 (0.72)
Total cholesterol (mmol/L) *	4.83 (0.84)	5.11 (1.10)	5.10 (0.64)	5.67 (0.95)	5.43 (1.34)	5.58 (1.23)
HDL-C (mmol/L) *	1.12 (0.30)	1.08 (0.31)	1.04 (0.22)	1.31 (0.30)	1.16 (0.31)	1.17 (0.31)
LDL-C (mmol/L)	3.46 (0.73)	2.75 (0.93)	3.38 (0.65)	3.82 (0.57)	3.09 (1.05)	3.75 (1.13)
AST (μkat/L) *	0.42 (0.09)	0.43 (0.13)	0.42 (0.12)	0.44 (0.12)	0.45 (0.16)	0.43 (0.13)
ALT (μkat/L) *	0.48 (0.15)	0.53 (0.27)	0.48 (0.22)	0.55 (0.29)	0.53 (0.19)	0.55 (0.33)
Glucose (mmol/L) *	5.43 (1.40)	5.99 (1.39)	6.09 (2.11)	5.45 (0.52)	5.11 (0.44)	5.59 (1.65)
HOMA index *	4.81 (7.37)	4.67 (6.05)	3.69 (2.75)	2.92 (1.76)	3.72 (1.65)	2.99 (1.89)

Control Diet based on CREA-AN guidelines (INRAN); Low Glycemic Index Mediterranean Diet (LGIMD); Physical Activity 1 based on the Aerobic Activity Program (ATFIS_1): Physical Activity 2 based on the combination of Aerobic Activity Program and Resistance Training (ATFIS_2); combination of LGIMD and ATFIS1 or ATFIS2 physical activity program (LGIMD-ATFIS_1; LGIMD-ATFIS_2); BMI: Body Mass Index; CAP: Controlled Attenuation Parameter; HDL-C: High-Density Lipoprotein Cholesterol; LDL-C: Low-Density Lipoprotein Cholesterol; AST: Aspartate Aminotransferase; and ALT: Alanine Aminotransferase. Cells showing the subjects’ characteristics contain * Mean (±SD). ** Number; percentages calculated per rows.

## Data Availability

The obtained 16S rRNA fastQ sequences are available from the NCBI Bioproject database. The project submitted entry refers to Submission ID: SUB11191038; BioProject ID: PRJNA816444.

## Supplementary Materials

The following supporting information can be downloaded at: https://www.mdpi.com/article/10.3390/nu14091773/s1, Qiime2 statistics and taxa relative abundances. Table S1: ‘metadata’ includes the metadata variables used to compute the downstream statistical comparisons. Table S2: ‘demux filtered statistics’ includes the total number of raw, retained, truncated, too short and maximum ambiguous bases exceeding reads. Table S3: ‘paired end demux stats’ reports the per-sample number of forward and reverse read counts. Table S4: ‘deblur stats’ reports the entire output removed by the Deblur software used for read denoizing. Tables S5–S10: report the taxa percentage abundances at the phylum, class, order, family, genus and species level, respectively. Table S11: Kruskal–Wallis (pairwise) test between the paired groups. Pairwise Kruskal–Wallis corrected for the multiple test (Benjamini–Hochberg). Faith’s PD index together with p- and multiple test corrected values (q-values). Figure S1: Trial flowchart. Experimental design reporting the number of patients excluded from the cohort, together with the per-group number of samples analyzed. Figure S2: Faith’s PD alpha diversity. Faith’s PD alpha-diversity metric was computed for each of the intervention groups. Figure S3: Principal component analyses based on genus abundances. PCA biplot of the variables and samples. The variable contribution of genera is reported as cos2 vectors. Arrow thickness and colors are indicative of the variable weight. Figure S4: DAPC resulted from the application of the find cluster function. Panel A) reports the Bayesian Information Criterion (BIC) curve, where the K-means are shown for the y axis and cluster number for the x axis. Panel B) DAPC based on find.cluster calculation. Eigen values are reported at the bottom-right of the figure. Figure S5: Boxplot distributions of taxa that contributed most to the separation of groups in the DAPC analysis. Over-threshold genera from the DAPC loading plot were singly inspected for their relative abundances and plotted as interquartile ranges. Figure S6: LGIMD vs. LGIMD-ATFIS_1 statistically significant predicted metabolic pathway. Two-sided corrected Welch test between the LGIMD and LGIMD-ATFIS_1 diet/physical activity intervention groups. Mean relative frequency, standard derivation, *p*-value and corrected *p*-value are reported for the only statistically significant differences between the two groups: the CMP–legionaminate biosynthesis 1 pathway.
