# Supplementary material for: A Low Glycemic Index Mediterranean Diet Combined with Aerobic Physical Activity Rearranges the Gut Microbiota Signature in NAFLD Patients
Source: Nutrients. 2022 Apr 23;14(9):1773. doi: 10.3390/nu14091773 (PMC9101735; doi:10.3390/nu14091773)
Supplement: Supplementary file 1 [file nutrients-14-01773-s001.zip › Supplementary Figure S5.pdf]

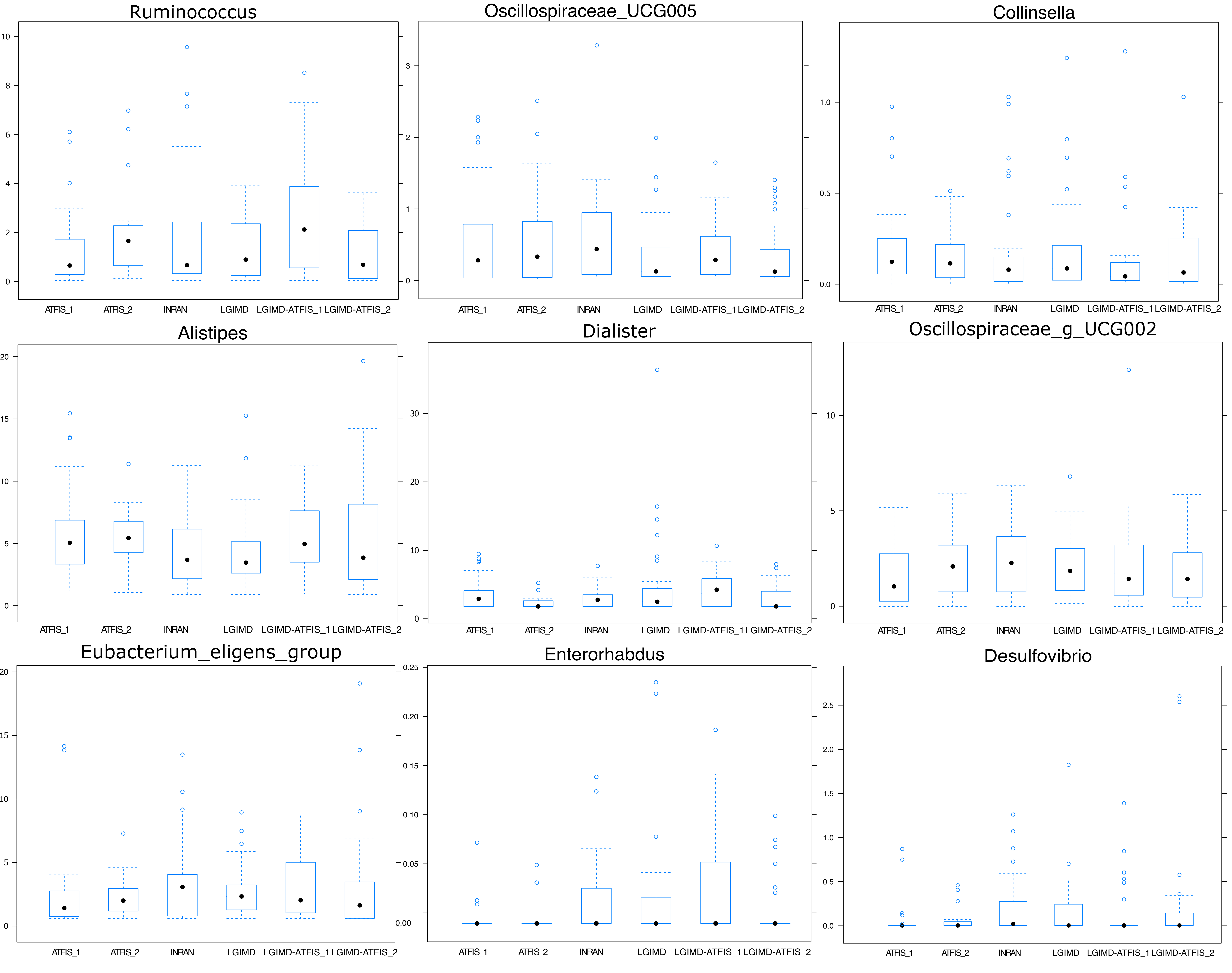
Ruminococcus
0
2
4
6
8
10
ATFIS_1 ATFIS_2 INRAN LGIMD LGIMD-ATFIS_1 LGIMD-ATFIS_2
Oscillospiraceae_UCG005
0
1
2
3
ATFIS_1 ATFIS_2 INRAN LGIMD LGIMD-ATFIS_1 LGIMD-ATFIS_2
Collinsella
0.0
0.5
1.0
ATFIS_1 ATFIS_2 INRAN LGIMD LGIMD-ATFIS_1 LGIMD-ATFIS_2
Alistipes
0
5
10
15
20
ATFIS_1 ATFIS_2 INRAN LGIMD LGIMD-ATFIS_1 LGIMD-ATFIS_2
Dialister
0
10
20
30
ATFIS_1 ATFIS_2 INRAN LGIMD LGIMD-ATFIS_1 LGIMD-ATFIS_2
Oscillospiraceae_g_UCG002
0
5
10
ATFIS_1 ATFIS_2 INRAN LGIMD LGIMD-ATFIS_1 LGIMD-ATFIS_2
Eubacterium_eligens_group
0
5
10
15
20
ATFIS_1 ATFIS_2 INRAN LGIMD LGIMD-ATFIS_1 LGIMD-ATFIS_2
Enterorhabdus
0.00
0.05
0.10
0.15
0.20
0.25
ATFIS_1 ATFIS_2 INRAN LGIMD LGIMD-ATFIS_1 LGIMD-ATFIS_2
Desulfovibrio
0.0
0.5
1.0
1.5
2.0
2.5
ATFIS_1 ATFIS_2 INRAN LGIMD LGIMD-ATFIS_1 LGIMD-ATFIS_2
