# Supplementary material for: A Low Glycemic Index Mediterranean Diet Combined with Aerobic Physical Activity Rearranges the Gut Microbiota Signature in NAFLD Patients
Source: Nutrients. 2022 Apr 23;14(9):1773. doi: 10.3390/nu14091773 (PMC9101735; doi:10.3390/nu14091773)
Supplement: Supplementary file 1 [file nutrients-14-01773-s001.zip › Supplementary Figure S6.pdf]

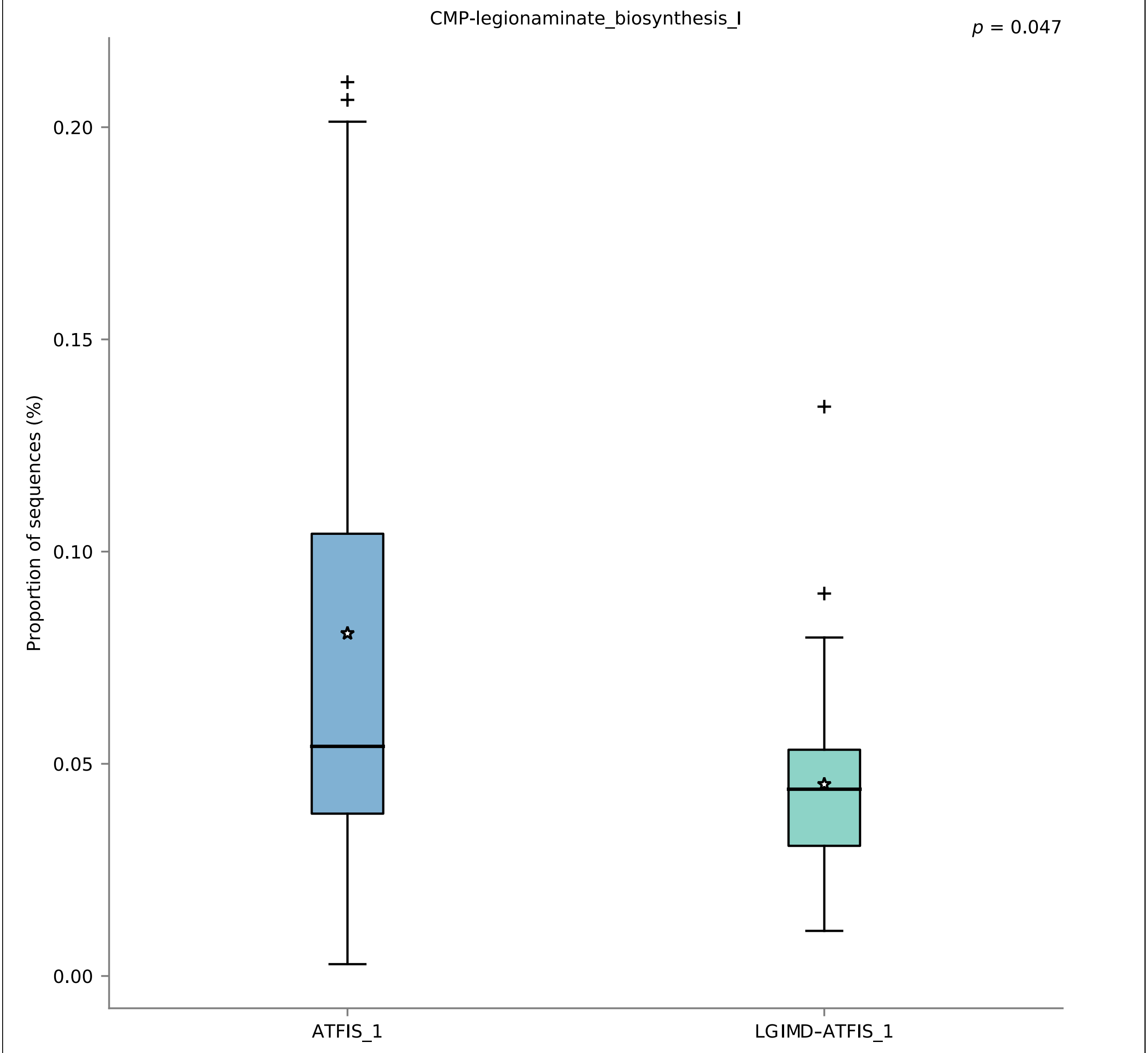

CMP-legionaminate_biosynthesis_I	values
LGIMD-ATFIS_1: mean rel. freq. (%)	0.04516236
LGIMD-ATFIS_1: std. dev. (%)	0.02327737
ATFIS_1: mean rel. freq. (%)	0.08072176
ATFIS_1: std. dev. (%)	0.05830324
p-values	0.00025869
p-values (corrected)	0.04695201
Difference between means	-0.0355594
95.0% lower CI	-0.0539378
95.0% upper CI	-0.0171811
