# Supplementary figures and images for: A Low Glycemic Index Mediterranean Diet Combined with Aerobic Physical Activity Rearranges the Gut Microbiota Signature in NAFLD Patients
Source: Nutrients. 2022 Apr 23;14(9):1773. doi: 10.3390/nu14091773 (PMC9101735; doi:10.3390/nu14091773)

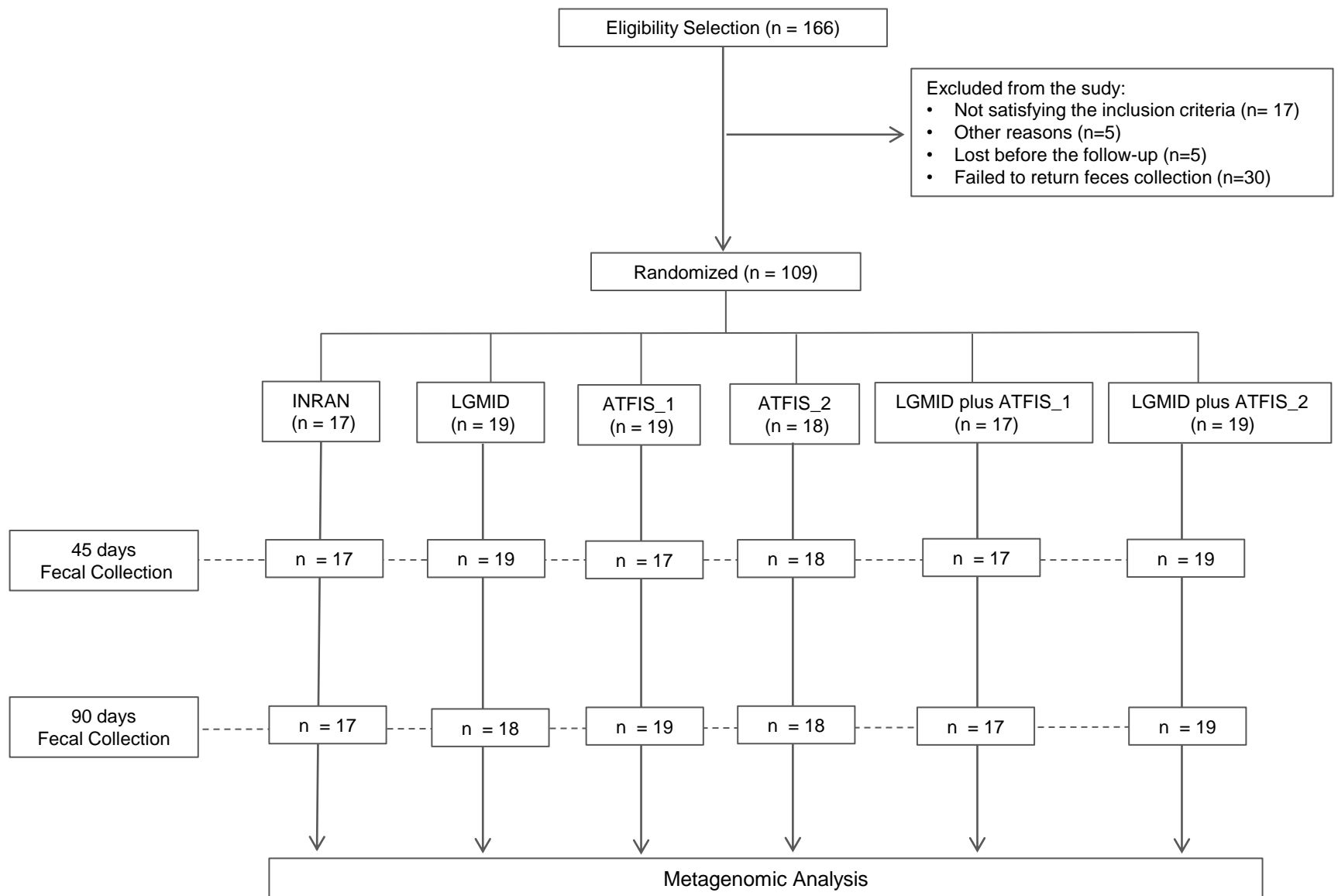

Supplement: Supplementary file 1 [file nutrients-14-01773-s001.zip › Supplementary Figure S1.pdf]

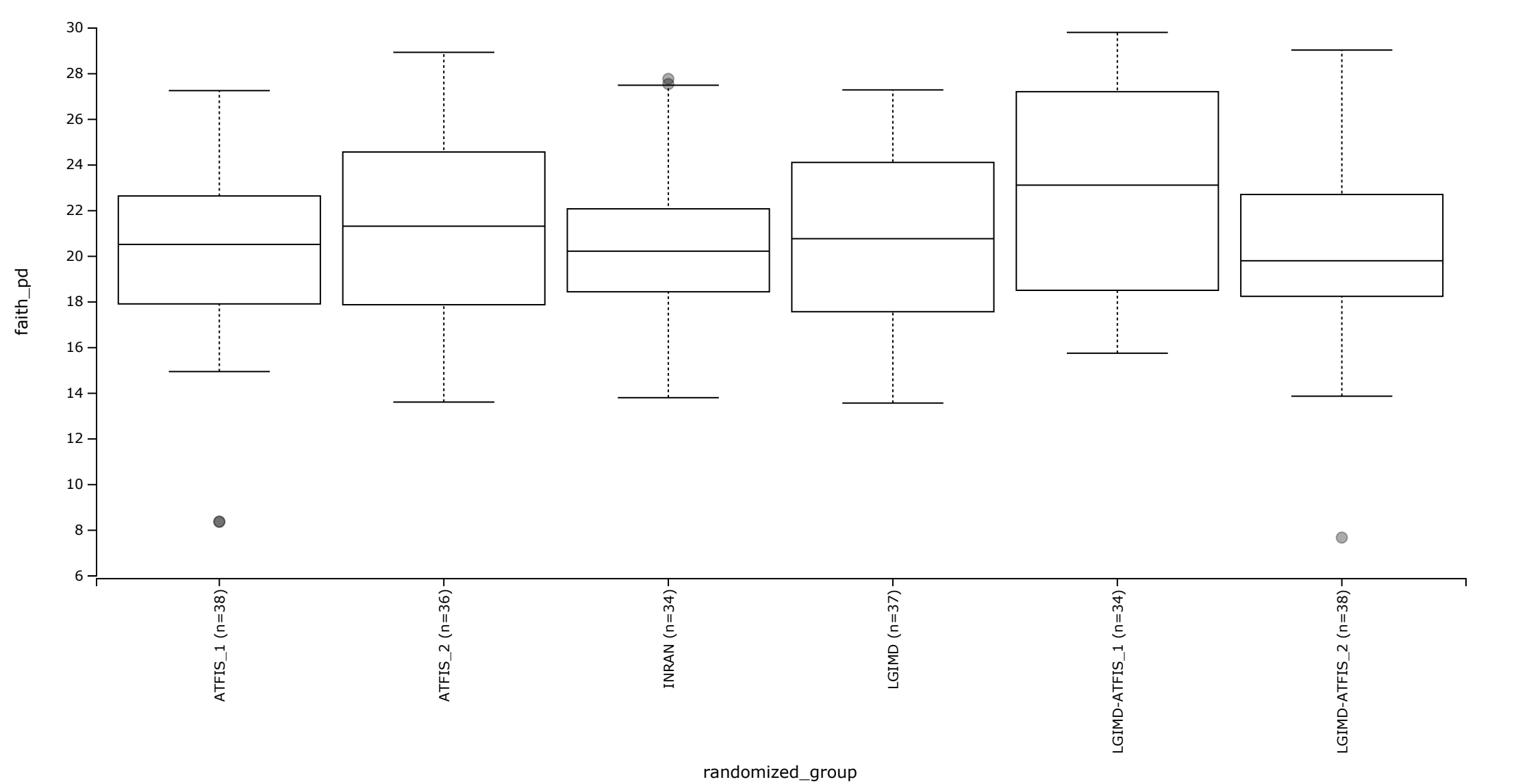

Supplement: Supplementary file 1 [file nutrients-14-01773-s001.zip › Supplementary Figure S2.pdf]

PCA – Biplot

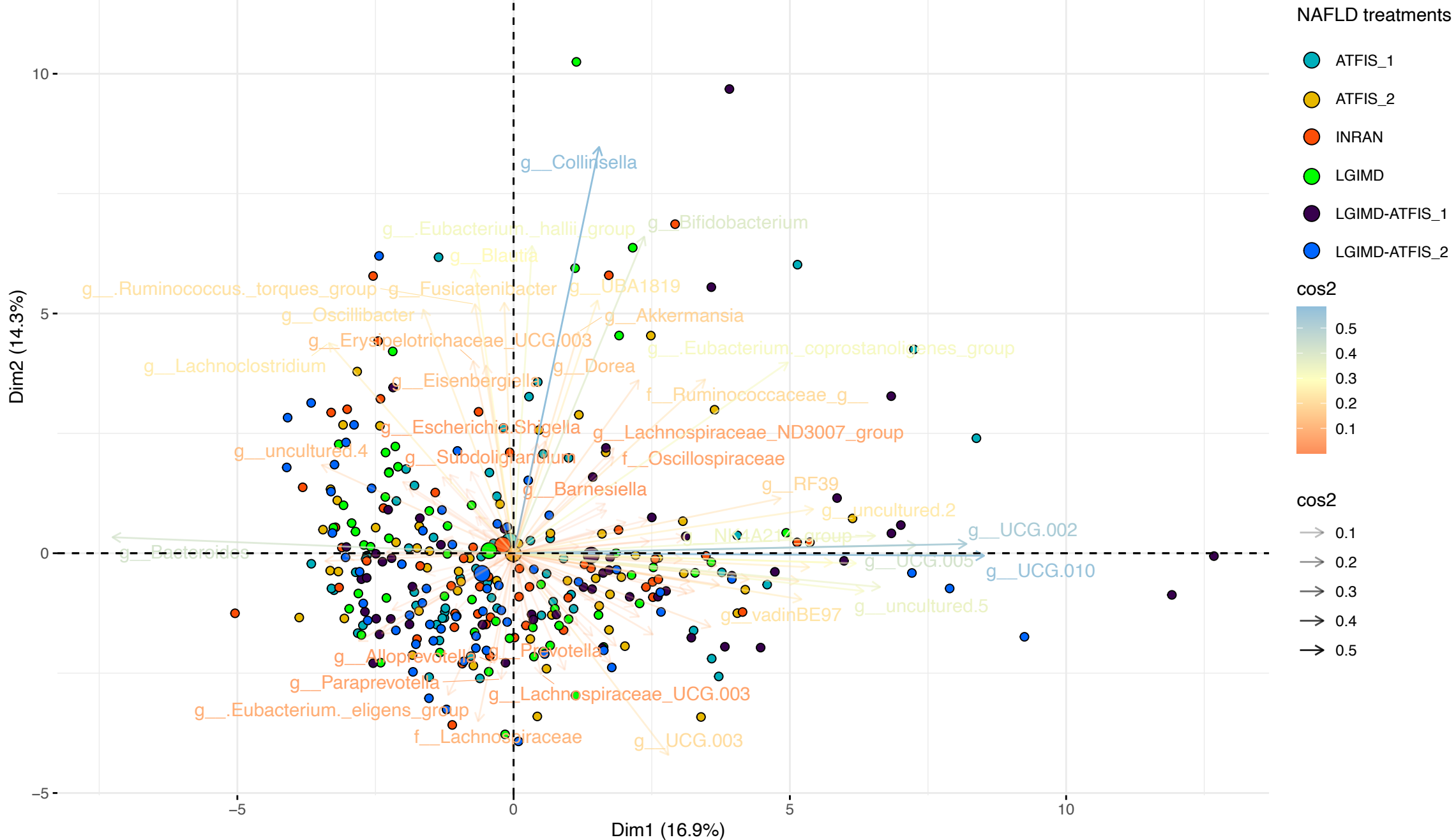

Supplement: Supplementary file 1 [file nutrients-14-01773-s001.zip › Supplementary Figure S3.pdf]

Value of BIC  
versus number of clusters

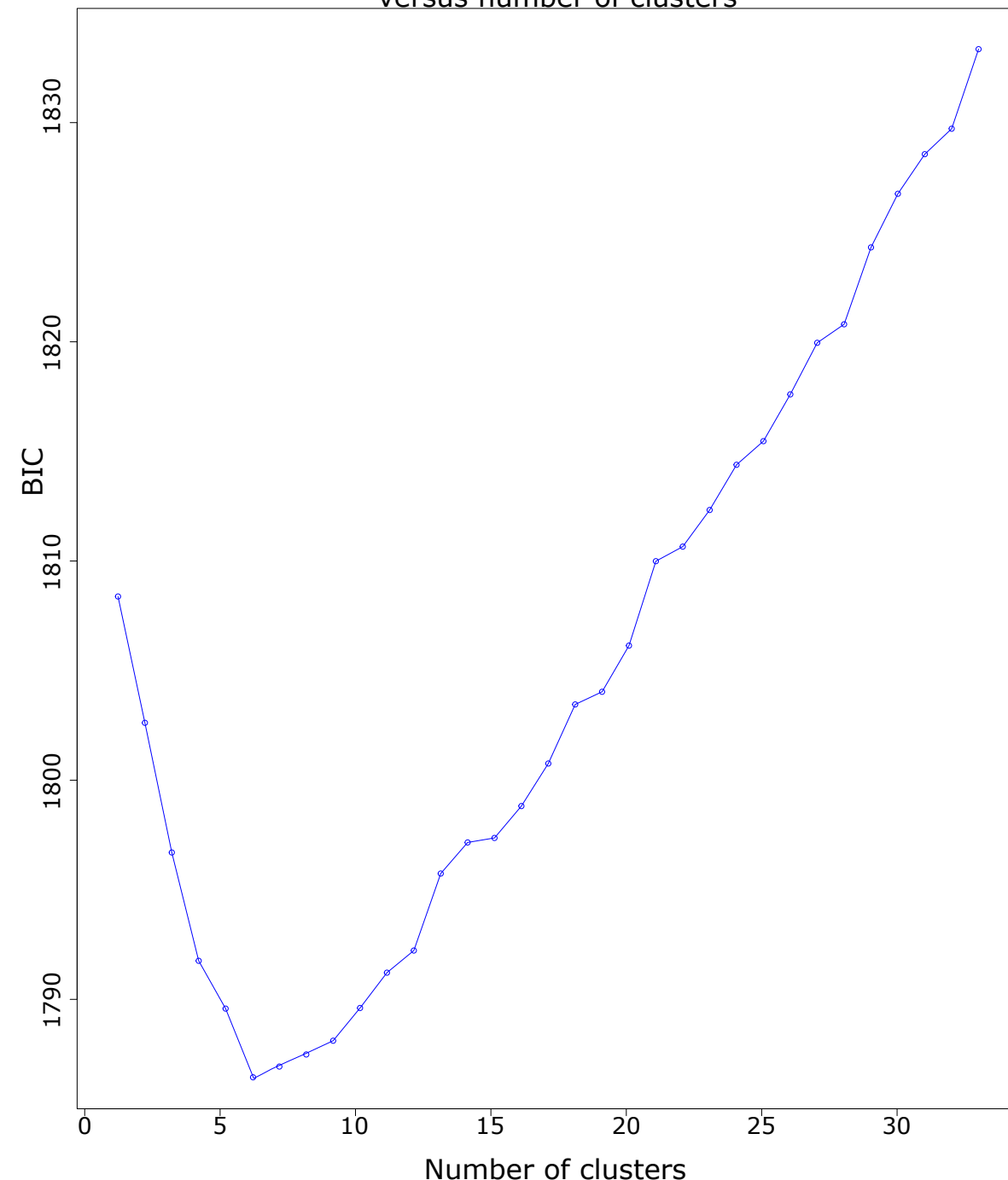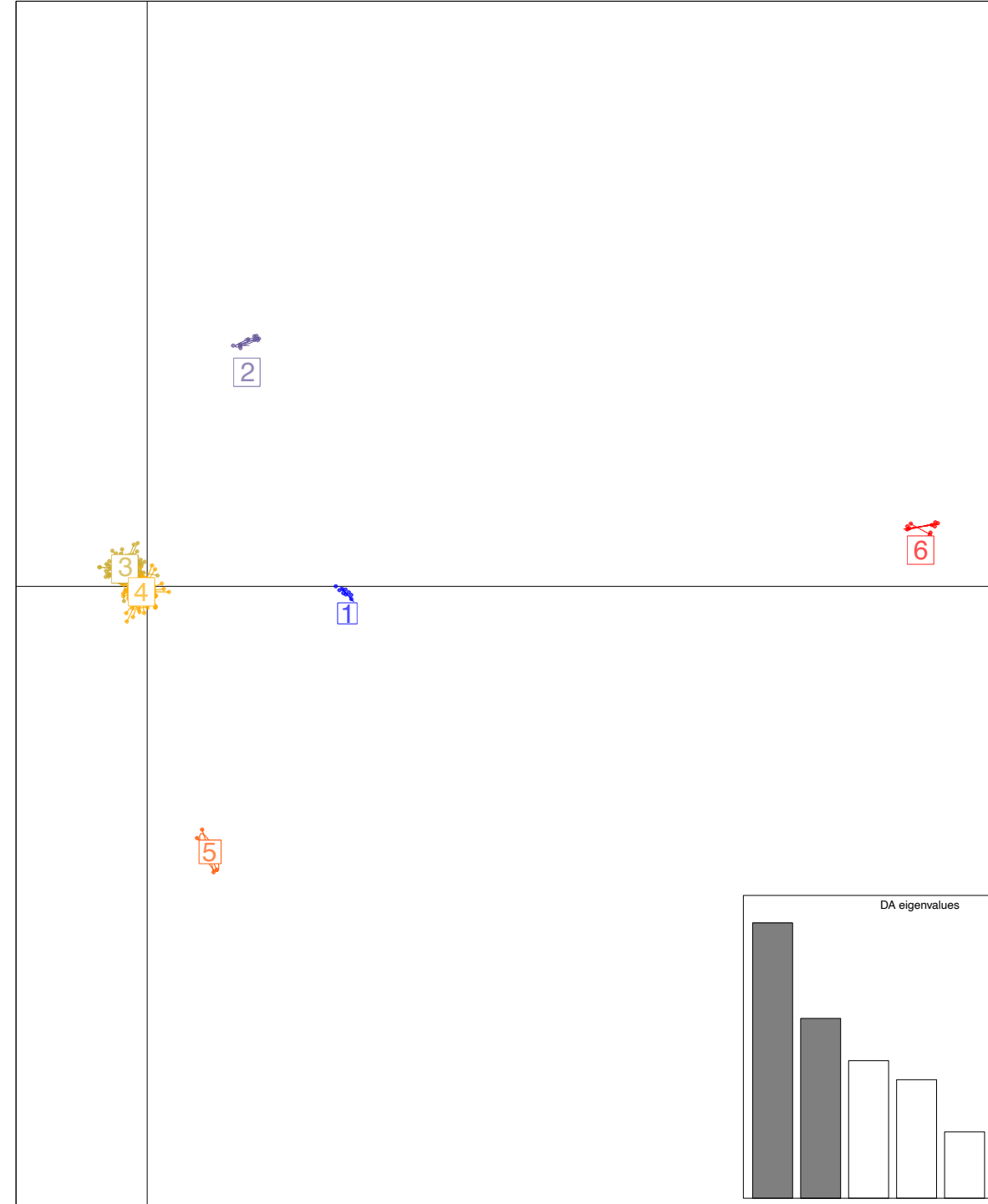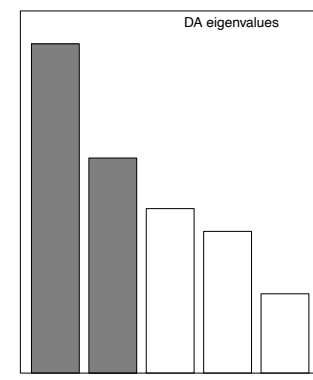

Supplement: Supplementary file 1 [file nutrients-14-01773-s001.zip › Supplementary Figure S4.pdf]
